# Can endotracheal bioimpedance cardiography assess hemodynamic response to passive leg raising following cardiac surgery?

**DOI:** 10.1186/2110-5820-2-26

**Published:** 2012-07-11

**Authors:** Jean-Luc Fellahi, Marc-Olivier Fischer, Audrey Dalbera, Massimo Massetti, Jean-Louis Gérard, Jean-Luc Hanouz

**Affiliations:** 1 Department of Anesthesiology and Critical Care Medicine, CHU de Caen, Caen, F-14000, France; 2 Univ Caen, Faculty of Medicine, Caen, F-14000, France; 3 Department of Cardiothoracic Surgery, CHU de Caen, Caen, F-14000, France

**Keywords:** Cardiac surgery, Cardiac monitoring, Bioimpedance cardiography, Passive leg raising, Fluid responsiveness

## Abstract

**Background:**

The utility of endotracheal bioimpedance cardiography (ECOM) has been scarcely reported. We tested the hypothesis that it could be an alternative to pulse contour analysis for cardiac index measurement and prediction in fluid responsiveness.

**Methods:**

Twenty-five consecutive adult patients admitted to the intensive care unit following conventional cardiac surgery were prospectively included and investigated at baseline, during passive leg raising, and after fluid challenge. Comparative cardiac index data points were collected from pulse contour analysis (CI_PC_) and ECOM (CI_ECOM_). Correlations were determined by linear regression. Bland-Altman analysis was used to compare the bias, precision, and limits of agreement. Percentage error was calculated. Changes in CI_PC_ (ΔCI_PC_) and CI_ECOM_ (ΔCI_ECOM_) during passive leg raising were collected to assess their discrimination in predicting fluid responsiveness.

**Results:**

A significant relationship was found between CI_PC_ and CI_ECOM_ (r = 0.45; *P* < 0.001). Bias, precision, and limits of agreement were 0.44 L.min^-1^.m^-2^ (95% confidence interval, 0.33-0.56), 0.59 L.min^-1^.m^-2^, and −0.73 to 1.62 L.min^-1^.m^-2^, respectively. Percentage error was 45%. A significant relationship was found between percent changes in CI_PC_ and CI_ECOM_ after fluid challenge (r = 0.42; *P* = 0.035). Areas under the ROC curves for ΔCI_PC_ and ΔCI_ECOM_ to predict fluid responsiveness were 0.72 (95% confidence interval, 0.5–0.88) and 0.81 (95% confidence interval, 0.61-0.94), respectively.

**Conclusions:**

ECOM is not interchangeable with pulse contour analysis but seems consistent to monitor cardiac index continuously and could help to predict fluid responsiveness by using passive leg raising.

## Background

The respiratory variations in arterial pulse pressure (PPV) or stroke volume (SVV) are widely recognized to guide fluid management [1,2]. However, it has been suggested that only 23% of the patients undergoing an anesthetic procedure met the criteria for the routine use of PPV [3], and the gray zone approach has shown that despite a strong predictive value, PPV may be inconclusive in approximately 25% of patients for prediction of fluid responsiveness during mechanical ventilation [4]. Passive leg raising (PLR) is a routinely applied bedside method that also predicts volume responsiveness [5-7]. In a recent meta-analysis, PLR was able to predict fluid responsiveness accurately regardless of the ventilation mode and the cardiac rhythm [8]. Its clinical application requires a continuous dynamic assessment of cardiac output, and changes in cardiac output during PLR have been found to predict fluid responsiveness accurately in postoperative cardiac surgery patients when uncalibrated pulse contour analysis or bioreactance-based NICOM system were used [9,10].

The Endotracheal Cardiac Output Monitor (ECOM, ConMed, Utica, NY) is a new Food and Drug Administration-approved device that provides continuous cardiac index measurement via a specifically designed endotracheal tube using three-dimensional bioimpedance in conjunction with an arterial catheter. The ECOM device was first evaluated in an animal study, which suggested this new technology was both promising and safe [11]. Since then, validation studies have been scarcely reported in humans [12-14]. These studies compared ECOM with either pulmonary artery [12,13] or transpulmonary [14] thermodilution and with transesophageal echocardiography [13] and found a poor correlation and lack of agreement in the setting of cardiac surgery. However, ECOM was convenient and consistent to monitor cardiac output continuously [13] and seemed able to track the direction of its changes under dynamic loading conditions [14]. Furthermore, SVV given by ECOM had the ability to predict fluid responsiveness with a good accuracy and discrimination [14]. No previous study focused on the comparison between ECOM and a continuous, mini-invasive, beat-to-beat cardiac output monitor, and the utility of ECOM to predict fluid responsiveness by means of PLR has never been evaluated.

Therefore, the objectives of the present study conducted in patients undergoing conventional cardiac surgery with cardiopulmonary bypass were twofold: 1) to compare continuous endotracheal bioimpedance cardiac index measured with ECOM with continuous calibrated pulse contour analysis measured with the PiCCO_2_ system; and 2) to assess the diagnostic accuracy of changes in cardiac index during PLR given by ECOM in predicting fluid responsiveness. We tested the hypothesis that the ECOM device would be a convenient and reliable tool for both cardiac index measurement and prediction in fluid responsiveness in that setting.

## Methods

### Patient population

This new study was conducted in accordance with the Statements for Reporting Studies of Diagnostic Accuracy (the STARD initiative) [15]. Twenty-five consecutive adult patients admitted to the cardiac surgical intensive care unit (ICU) following conventional cardiac surgery with cardiopulmonary bypass were investigated at the Teaching University Hospital of Caen (Caen, France) from January to June 2011. Institutional approval was obtained from the Ethical Committee (Comité de Protection des Personnes Nord Ouest III, CHU, avenue de la Côte de Nacre, BP 95182, 14033 Caen Cedex 9, France; Chairperson Pr Claude Bazin) on July 15, 2010. Because data were collected during routine care that conformed to standard procedures currently used in our institution, authorization was granted to waive written, informed consent (Ref. CPP: A10-D16-VOL.10). All patients scheduled for conventional cardiac surgery with cardiopulmonary bypass (coronary artery bypass grafting, aortic, and/or mitral valve replacement or repair and combined cardiac surgery) and requiring advanced hemodynamic monitoring during a 6-month period were eligible for the study. They were enrolled if they received fluid challenge during the initial postoperative period, according to the decision of the attending physician. Patients undergoing emergency surgery (less than 24 h), redo surgery, off-pump coronary artery bypass grafting and complex, unusual procedures, or not requiring advanced hemodynamic monitoring were not included in the study. Patients with a prior history of allergy to hydroxyethyl starch also were not included in the study.

### Perioperative management

General anesthesia and postoperative management followed institutional standards. During the postoperative period, all patients were admitted to the ICU and extubated after the completion of the institutional weaning protocol. At the time of the study, they were intubated, ventilated (volume-controlled regimen), and sedated with propofol and remifentanil to maintain a Ramsay score >5. For each patient, a radial artery catheter (Leadercath 20 G, Vygon, Ecouen, France), a jugular central venous catheter, and a femoral 5-F thermistor-tipped arterial catheter (Pulsiocath™ thermodilution catheter PV2015L20N [Pulsion France sarl, La Montagne, France]) were inserted in the operating room after induction of general anesthesia [14]. The Pulsiocath thermodilution catheter was connected to the stand-alone PiCCO_2_ computer PC8500 version 2.0 (Pulsion Medical Systems, Munich, Germany). Continuous pulse contour cardiac index measurement was initiated after the initial calibration of the system by a triplicate 15-mL ice-cold normal saline injection through the central venous catheter (transpulmonary thermodilution) [16]. The calibration process was then repeated at the arrival in the ICU and before and after fluid challenge in the ICU. All patients were intubated with a 7.5-mm ECOM endotracheal tube (ECOM-ETT 7.5 G, ConMed, Utica, NY) after the induction of general anesthesia. This endotracheal tube is specially designed and contains seven silver electrodes on the cuff and tube that continuously measure the bioimpedance signal from the ascending aorta, in close proximity to the trachea [11]. After processing, it provides real-time continuous stroke volume and cardiac index values. At the arrival in ICU, the ECOM pressure monitor was connected to the radial arterial line and then to the ECOM endotracheal tube impedance wires. All pressure monitors were zeroed at the midaxillary line. Intra- and postoperative hemodynamic management were left to the discretion of the attending anesthesiologist, who was not involved in the study protocol and was unaware of the hemodynamic data given by the ECOM system. Hemodynamic therapy in the ICU was guided by usual clinical parameters and values obtained from continuous mean arterial pressure and calibrated pulse contour monitoring. Intravenous fluids and vasoactive drugs were used as appropriate. During the brief observation period, ventilator settings, sedation, and vasoactive drugs were continued unchanged.

### Study protocol

The patient was enrolled after the decision by the attending physician to administer a fluid challenge (500 mL of hydroxyethyl starch 130/0.4, 6% over 15 minutes) within the first 6 postoperative hours. Four consecutive sets of measurement were recorded for each patient: 1) at baseline in the 45° semirecumbent position; 2) during a 1-minute PLR, which consisted of simply pivoting the entire bed by the automatic pivotal motion, as previously described [17]; 3) at return to baseline in the 45° semirecumbent position; and 4) 10 minutes after fluid challenge as described above. At each step, simultaneous comparative cardiac index data points were collected from calibrated pulse contour analysis (CI_PC_) and ECOM (CI_ECOM_). CI_PC_ was used to define the positive response to fluid challenge as an increase of at least 15%. Changes in CI_PC_ (ΔCI_PC_) and CI_ECOM_ (ΔCI_ECOM_) during PLR were collected to assess the comparative discrimination of both dynamic indices in predicting fluid responsiveness.

### Statistical analysis

On the basis of the literature and our previous reports [14,18], the number of patients was empirically fixed at 25. Data are expressed as mean ± SD or median [extremes] for nonnormally distributed variables (Kolmogorov-Smirnov test) or number and percentage as appropriate. Continuous variables were analyzed with the unpaired Student *t* and Mann-Whitney *U* tests according to their distribution. Absolute values and changes in hemodynamic parameters after fluid challenge were compared by using the paired Wilcoxon’s test. ANOVA (two-factor study with repeated measures on one factor) was used to compare changes in CI_PC_ and CI_ECOM_ after PLR and fluid challenge in responders and nonresponders. Correlations between absolute values of CI_PC_ and CI_ECOM_ and between percent changes in cardiac index (measured with both PiCCO_2_ and ECOM) when fluid challenge was applied were determined by linear regression. Bland-Altman analysis was used to compare the bias, precision (SD of bias), and limits of agreement (bias ± 1.96 SD) of CI_PC_ versus CI_ECOM_. Because we performed multiple measurements in the same patients, we replaced the classic Bland-Altman analysis [19] by a specific technique dedicated to the evaluation of the agreement between methods of measurement with multiple observations per individual [20]. Percentage error to determine acceptable limits of agreement between both techniques of cardiac index measurement was calculated using the formula given by Critchley and Critchley [21]. To assess the discrimination of ΔCI_PC_ and ΔCI_ECOM_ during PLR in predicting fluid responsiveness, we determined the empiric receiver operating characteristic (ROC) curves and calculated the areas under the ROC curves and their 95% confidence interval. Comparison of areas under the ROC curve was performed by using a nonparametric paired technique, as described previously [22]. The ROC curves also were used to determine the optimal thresholds for ΔCI_PC_ and ΔCI_ECOM_ to predict fluid responsiveness. The optimal threshold was the value that maximized the sum of the sensitivity and specificity. Assessment of the diagnostic performances of an increased ΔCI_PC_ or ΔCI_ECOM_ above the threshold value in predicting fluid responsiveness was performed by calculating the sensitivity, specificity, positive, and negative likelihood ratios and their 95% confidence interval values.

*P* < 0.05 was considered statistically significant, and all *P* values were two-tailed. Statistical analyses were performed by using MedCalc® Software bvba version 12.2.1 (Mariakerke, Belgium).

## Results

**Figure 1:**
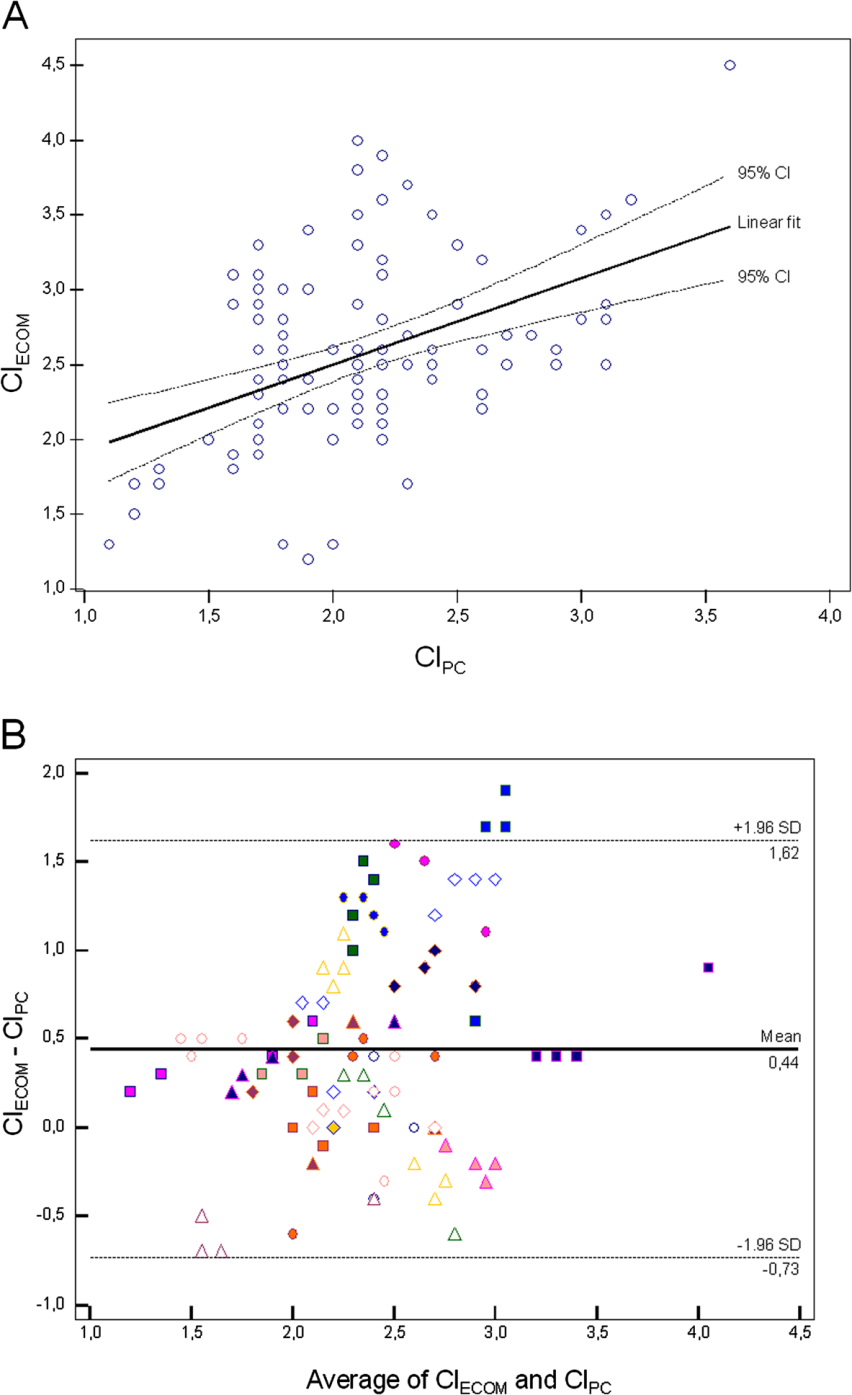
**Relationship between CI**_**PC**_**and CI**_**ECOM**_**in 25 patients (100 paired data points).** The linear fit is given with 95% confidence interval (**A**); Bland-Altman analysis between CI_PC_ and CI_ECOM_ in 25 patients (100 paired data points). The mean bias is given with its limits of agreement (**B**). CI_ECOM_ = cardiac index determination using ECOM (L.min^-1^.m^-2^); CI_PC_ = cardiac index determination using pulse contour analysis (L.min^-1^.m^-2^).

Patients’ baseline characteristics are indicated in Table 1. Fourteen (56%) patients experienced an increase in CI_PC_ of at least 15% following fluid challenge and were subsequently classified as responders, according to the primary definition. Eleven (44%) patients were nonresponders. CI_PC_ ranged from 1.1 to 3.6 L.min^-1^.m^-2^ and CI_ECOM_ from 1.2 to 4.5 L.min^-1^.m^-2^ (2.1 ± 0.5 vs. 2.6 ± 0.6, *P* < 0.001). We observed a weak but statistically significant relationship between absolute values of CI_PC_ and CI_ECOM_ (r = 0.45; *P* < 0.001; Figures 1A). Bias between CI_PC_ and CI_ECOM_ was 0.44 L.min^-1^.m^-2^ (95% confidence interval, 0.33-0.56), precision was 0.59 L.min^-1^.m^-2^, and limits of agreement were −0.73 to 1.62 L.min^-1^.m^-2^ (±1.17 L.min^-1^.m^-2^; Figures 1B). Percentage error between pulse contour analysis and ECOM for cardiac index measurement was 45%.

**Table 1:** Patients baseline characteristics (N = 25)

**Age (yr)**	**64 ± 13**
Sex (M/F)	17/8
Body mass index (kg/m^2^)	27.1 ± 6.0
EuroSCORE	3 [2-13]
Preoperative left ventricular ejection fraction (%)	68 ± 9
Hypertension	11 (44)
Peripheral vascular disease	7 (28)
Coronary artery disease	16 (64)
Diabetes mellitus	3 (12)
Type of surgery	
Coronary artery bypass grafting	9 (36)
Aortic valve replacement	8 (32)
Mitral valve repair	2 (8)
Combined cardiac surgery	6 (24)
Mechanical ventilation	
Inspired fraction of O_2_ (%)	51 ± 6
Tidal volume/ideal body weight (mL/kg)	9.0 ± 1.2
Respiratory rate (per min)	13 ± 2
Positive end-expiratory pressure (cmH_2_O)	4.6 ± 1.4
Inotropic or vasoactive requirement	
Dobutamine^a^	1 (4)
Norepinephrine^b^	7 (28)
Central core temperature (°C)	36.4 ± 0.7

Values are mean ± SD or median [extremes] or number (%).

^a^15 μg/kg/min; ^b^from 0.02 to 0.23 μg/kg/min.

**Figure 2:**
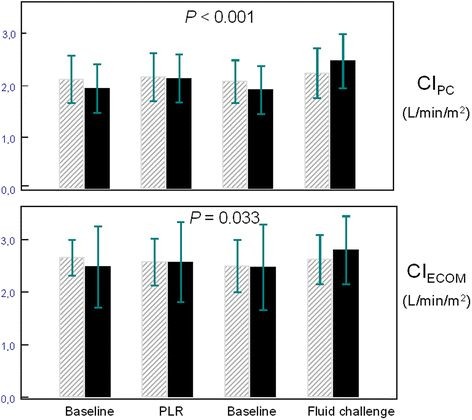
**CI**_**PC**_**and CI**_**ECOM**_**at baseline, during passive leg raising and after fluid challenge in responders (black boxes) and non responders (striated grey boxes).** Values are mean ± SD. *P* value refers to ANOVA (two-factor study with repeated measures on one factor). CI_ECOM_ = cardiac index determination using ECOM (L.min^-1^.m^-2^); CI_PC_ = cardiac index determination using pulse contour analysis (L.min^-1^.m^-2^); PLR = passive leg raising.

**Figure 3:**
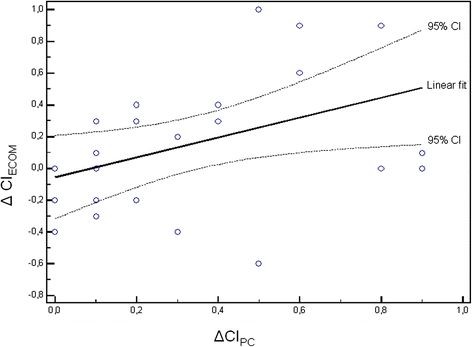
**Relationship between percent changes in cardiac index determination using ECOM (ΔCI**_**PC**_**) and cardiac index determination using pulse contour analysis (ΔCI**_**ECOM**_**) following fluid challenge in 25 patients (25 paired data points).** The linear fit is given with 95% confidence interval.

Hemodynamic data at baseline and after fluid challenge in responders and nonresponders are indicated in Table 2. The ECOM signal quality index was excellent at any time in all patients, indicating that measurements of cardiac index were valuable for analysis. Whereas CI_PC_ was significantly increased in both responders and nonresponders, CI_ECOM_ only increased in responders (Table 2). In the whole cohort of patients, fluid challenge induced a significant increase in CI_PC_ by 20% (from 2.0 ± 0.5 L.min^-1^.m^-2^ to 2.4 ± 0.5 L.min^-1^.m^-2^; *P* < 0.001), whereas the increase in CI_ECOM_ was only 8% (from 2.5 ± 0.6 L.min^-1^.m^-2^ to 2.7 ± 0.6 L.min^-1^.m^-2^; *P* = 0.449). PLR induced a significant increase in CI_PC_ by 10% (from 2.0 ± 0.5 L.min^-1^.m^-2^ to 2.2 ± 0.5 L.min^-1^.m^-2^; *P* = 0.017), whereas the increase in CI_ECOM_ was only 4% (from 2.5 ± 0.6 L.min^-1^.m^-2^ to 2.6 ± 0.6 L.min^-1^.m^-2^; *P* = 1.000). Changes in CI_PC_ and CI_ECOM_ during passive leg raising and after fluid challenge in responders and nonresponders are depicted in Figures 2. A weak but statistically significant positive relationship was found between percent changes in CI_PC_ and CI_ECOM_ following fluid challenge (r = 0.42; *P* = 0.035; Figures 3).

**Table 2:** Hemodynamic data at baseline and after fluid challenge

	**Baseline**	**Fluid challenge**	***P*value**
MAP (mmHg)			
Responders (n = 14)	66 ± 10	76 ± 16	0.008
Non responders (n = 11)	59 ± 7	62 ± 11	0.322
Heart rate (beats/min)			
Responders (n = 14)	71 ± 15	71 ± 13	0.839
Non responders (n = 11)	71 ± 15	66 ± 13	0.002
CI_PC_ (L/min/m^2^)			
Responders (n = 14)	1.9 ± 0.5	2.5 ± 0.5	<0.001
Non responders (n = 11)	2.1 ± 0.5	2.2 ± 0.5	0.016
CI_ECOM_ (L/min/m^2^)			
Responders (n = 14)	2.5 ± 0.8	2.8 ± 0.6	0.024
Non responders (n = 11)	2.7 ± 0.3	2.6 ± 0.5	0.652
ScvO_2_ (%)			
Responders (n = 14)	65 ± 10	73 ± 7	<0.001
Non responders (n = 11)	63 ± 7	65 ± 6	0.102
Hemoglobin (g/dL)			
Responders (n = 14)	11.2 ± 1.3	9.8 ± 1.2	<0.001
Non responders (n = 11)	11.5 ± 1.8	10.3 ± 1.5	0.001
CVP (mmHg)			
Responders (n = 14)	5 ± 2	7 ± 3	0.027
Non responders (n = 11)	6 ± 4	8 ± 3	0.048
GEDV (mL/m^2^)			
Responders (n = 14)	583 ± 120	644 ± 101	<0.001
Non responders (n = 11)	730 ± 243	760 ± 268	0.102

Values are mean ± SD.

*CI*_*ECOM*_ = cardiac index ECOM; *CI*_*PC*_ = cardiac index pulse contour analysis; *CVP* = central venous pressure; *GEDV* = indexed global end-diastolic volume; *MAP* = mean arterial pressure; *ScvO*_*2*_ = central venous oxygen saturation.

**Figure 4:**
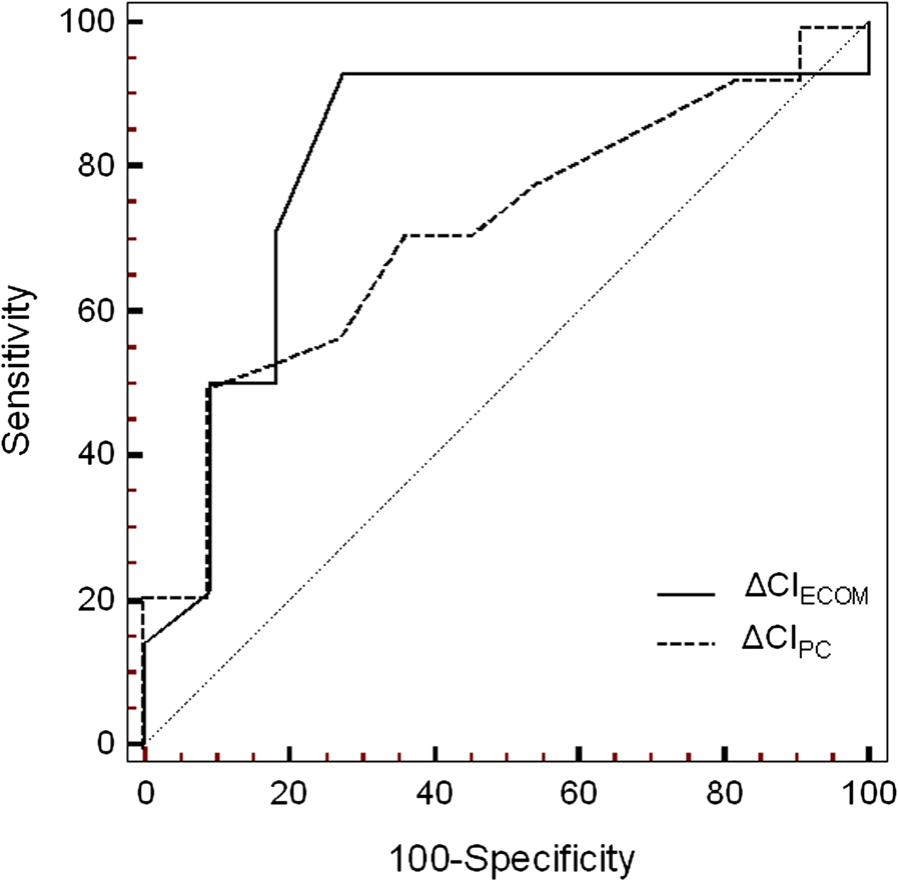
**ROC curves showing the relationship between sensitivity and specificity in determining the discrimination of ΔCI**_**PC**_**and ΔCI**_**ECOM**_**in predicting fluid responsiveness.** The dotted diagonal line is the no-discrimination curve. No significant difference was found between ROC curves. ΔCI_ECOM_ = change in cardiac index ECOM during passive leg raising; ΔCI_PC_ = change in cardiac index pulse contour analysis during passive leg raising; ROC = receiver operating characteristic curve.

Threshold values and the diagnostic performances of an elevated ΔCI_PC_ and ΔCI_ECOM_ in predicting fluid responsiveness are indicated in Table 3. An increase in CI_ECOM_ by 3% following PLR predicted fluid responsiveness with a sensitivity of 93% and a specificity of 73%. No significant difference was found among areas under the ROC curves for ΔCI_PC_ and ΔCI_ECOM_ (Figures 4).

**Table 3:** **Diagnostic performances of ΔCI**_**PC**_**and ΔCI**_**ECOM**_**in predicting fluid responsiveness**

	**ΔCI_PC_**	**ΔCI_ECOM_**
ROC_AUC_	0.72 (0.50-0.88)	0.81 (0.61-0.94)
Cutoff value (%)	6	3
Sensitivity	50 (23-77)	93 (66-100)
Specificity	91 (59-100)	73 (39-94)
Positive likelihood ratio	5.5 (3.2-9.6)	3.4 (2.3-5.0)
Negative likelihood ratio	0.6 (0.1-3.8)	0.1 (0.0-0.8)

Values are given with 95% confidence interval.

*ΔCI*_*ECOM*_ = change in cardiac index ECOM during passive leg raising; *ΔCI*_*PC*_ = change in cardiac index pulse contour analysis during passive leg raising; *ROC*_*AUC*_ = area under the receiver operated characteristics curve.

## Discussion

The main findings of the present study conducted in adult cardiac surgical patients are that: 1) the ECOM device, although less sensitive and not interchangeable with calibrated pulse contour analysis, provides consistent continuous measurements of cardiac index under dynamic conditions; and 2) changes in CI_ECOM_ during PLR predict fluid responsiveness with a good discrimination and could be a valuable alternative to calibrated pulse contour analysis in postoperative cardiac surgery patients.

Numerous previous published studies report a poor correlation and lack of agreement between cardiac output measured by usual thoracic electrical bioimpedance and a reference technique (most often thermodilution) in various subgroups of subjects [18,23-25]. Because of the anatomical proximity of the trachea and the ascending aorta, results could be improved markedly with the ECOM device, as initially demonstrated by Wallace et al. [11]. A poor correlation and lack of agreement between ECOM and pulmonary artery thermodilution at different intraoperative time points were, however, recently reported in patients undergoing cardiac surgery [12,13]. We further demonstrated that neither ECOM nor calibrated pulse contour analysis were interchangeable with transpulmonary thermodilution in the cardiac surgical setting [14]. In the present study, we found a weak positive relationship between both absolute values and percent changes in cardiac index when simultaneously using ECOM and pulse contour analysis in patients receiving volume loading. Even if an acceptable bias was evidenced, the limits of agreement were large and the percentage error as high as 45%. In their recent meta-analysis, Peyton and Chong [26] showed that none of the four alternative tested methods (i.e., pulse contour analysis, esophageal Doppler, partial P_CO2_ rebreathing, and thoracic electrical bioimpedance) achieved agreement with bolus thermodilution, which meets the expected 30% limits. They raise questions about the appropriateness of imposing arbitrary limits on the acceptability of accuracy and precision of cardiac index measurement, suggesting that the percentage error of agreement was only one marker of acceptability of a method. Thus, a more dynamic approach could be more interesting for clinical practice [27] and the efficacy of a clinical cardiac output monitor involves many factors other than its absolute accuracy and includes safety, convenience, and adaptability, which are characteristics that could be attributed to the ECOM system. Finally, a real-time tracking of the direction of changes in cardiac index could be more important than the ability of the monitor to deliver a highly accurate single measurement under stable conditions [28-30].

Numerous limitations that may decrease the applicability of PPV and/or SVV in daily clinical practice have been described in critically ill patients. This last point emphasizes the specific interest of PLR to predict fluid responsiveness in ICU [5-7], especially under conditions where heart-lung interaction indices cannot be interpretable [17]. Its clinical application requires a continuous and dynamic assessment of cardiac output. Changes in cardiac output during PLR have been found recently to predict fluid responsiveness accurately in postoperative cardiac surgery patients when uncalibrated pulse contour analysis was used [9]. We partially confirm these results, as an increase by 10% on average in cardiac index during PLR predicted fluid responsiveness with a moderate discrimination (ROC_AUC_ = 0.72). Minor changes in cardiac index during PLR when ECOM was used simultaneously predicted a positive response to fluid challenge with a ROC_AUC_ above 0.8. An explanation could be that PLR induces not only a brief and completely reversible self-volume challenge corresponding to the transfer of approximately 150 mL of blood [31] but also significant acute changes in arterial compliance that could lead to an increase in pulse contour-related stroke volume calculation. The reliability of the pulse contour method in detecting true variations in cardiac output would be negatively impacted by PLR itself. In contrast, ECOM, although clearly less sensitive than pulse contour analysis to detect changes in cardiac index during PLR, could be more reliable to predict fluid responsiveness. These last results are consistent with a previous report conducted in patients undergoing cardiac surgery and showing that the bioreactance-based NICOM system was clinically valid to predict fluid responsiveness from changes in cardiac output during PLR [10]. The cutoff value of 3% must be taken with caution, because we did not assess the reproducibility of ECOM in detecting changes in cardiac index after fluid challenge. To date, such a small value is lower than the reproducibility of all existing cardiac output measurement techniques. Thus, even if the present results suggest that ECOM could be a helpful monitor to conduct perioperative hemodynamic goal-oriented therapy in cardiac surgery patients requiring initial intubation, clinical utility/outcome (phase 3) studies are mandatory to further evaluate the ECOM device and definitely validate its clinical interest for routine practice [30].

Some comments are necessary concerning the limitations of the current study. First, we only investigated a small cohort of patients with a narrow range of cardiac index values. Other studies conducted in different subgroups of critical care patients with larger ranges of cardiac index values and testing different clinical approaches of variations in cardiac index are mandatory to validate our results externally. More severe patients with acute circulatory failure or shock states should probably be included in these studies. A greater sensitivity of ECOM could indeed be observed in these patients as previous reports using thoracic electrical bioimpedance suggest that the more the changes in preload are important, the more the magnitude of dZ/dt max (and subsequently CI values) is increased. Second, we only investigated sedated and ventilated patients with stable postoperative sinus rhythm. Future studies should evaluate the ability of ECOM to predict fluid responsiveness from changes in cardiac output during PLR in spontaneously breathing patients and/or in patients with cardiac arrhythmias, i.e., under clinical conditions where heart-lung interaction indices cannot be interpretable. Third, ECOM measurements, in their current form, are heavily dependent upon the fidelity of the arterial line tracing. Many patients after cardiac surgery have dampened arterial line waveforms. Even if the ECOM signal quality index was excellent at any time in all patients, indicating that measurements of cardiac index were valuable for analysis, we cannot exclude that this could affect the accuracy of the measurements from the ECOM device. Last, we used the first version of the ECOM software. Upgraded versions could give better results in the future.

## Conclusions

The ECOM device, although less sensitive and not interchangeable with calibrated pulse contour analysis, seems consistent to monitor cardiac index continuously and could track the direction of its changes under dynamic loading conditions in a safe and convenient manner in patients requiring both an endotracheal tube and advanced hemodynamic monitoring. The potential ability to predict fluid responsiveness with a good discrimination by using changes in cardiac index during PLR, if confirmed, could help to conduct perioperative hemodynamic goal-oriented therapy after cardiac surgery.

## Competing interests

The author(s) declare that they have no competing interests.

## Authors’ contributions

JLF conceived the study, participated in its design and coordination, performed statistical analysis and interpretation of the data, and wrote the manuscript. MOF participated in the coordination of the study, performed acquisition of data, and helped to write the manuscript. AD performed acquisition of data. MM, JLG, and JLH participated in the design and coordination of the study. All authors have read and approved the final manuscript.
